# Evolutionary patchwork of an insecticidal toxin shared between plant-associated pseudomonads and the insect pathogens *Photorhabdus* and *Xenorhabdus*

**DOI:** 10.1186/s12864-015-1763-2

**Published:** 2015-08-16

**Authors:** Beat Ruffner, Maria Péchy-Tarr, Monica Höfte, Guido Bloemberg, Jürg Grunder, Christoph Keel, Monika Maurhofer

**Affiliations:** Pathology, Institute of Integrative Biology, ETH Zurich, Universitätstrasse 2, CH-8092 Zurich, Switzerland; Department of Fundamental Microbiology, University of Lausanne, Biophore Building, CH-1015 Lausanne, Switzerland; Laboratory of Phytopathology, Department of Crop Protection, Ghent University, Ghent, Belgium; Institute of Medical Microbiology, University of Zurich, Zurich, Switzerland; Natural Resources Sciences, University of Applied Sciences ZHAW, Wädenswil, Switzerland

**Keywords:** *Pseudomonas*, *Photorhabdus* and *Xenorhabdus*, Insecticidal activity, Toxin evolution

## Abstract

**Background:**

Root-colonizing fluorescent pseudomonads are known for their excellent abilities to protect plants against soil-borne fungal pathogens. Some of these bacteria produce an insecticidal toxin (Fit) suggesting that they may exploit insect hosts as a secondary niche. However, the ecological relevance of insect toxicity and the mechanisms driving the evolution of toxin production remain puzzling.

**Results:**

Screening a large collection of plant-associated pseudomonads for insecticidal activity and presence of the Fit toxin revealed that Fit is highly indicative of insecticidal activity and predicts that *Pseudomonas protegens* and *P. chlororaphis* are exclusive Fit producers. A comparative evolutionary analysis of Fit toxin-producing *Pseudomonas* including the insect-pathogenic bacteria *Photorhabdus* and *Xenorhadus,* which produce the Fit related Mcf toxin, showed that *fit* genes are part of a dynamic genomic region with substantial presence/absence polymorphism and local variation in GC base composition. The patchy distribution and phylogenetic incongruence of *fit* genes indicate that the Fit cluster evolved via horizontal transfer, followed by functional integration of vertically transmitted genes, generating a unique *Pseudomonas*-specific insect toxin cluster.

**Conclusions:**

Our findings suggest that multiple independent evolutionary events led to formation of at least three versions of the Mcf/Fit toxin highlighting the dynamic nature of insect toxin evolution.

**Electronic supplementary material:**

The online version of this article (doi:10.1186/s12864-015-1763-2) contains supplementary material, which is available to authorized users.

## Background

Bacteria belonging to the *Pseudomonas fluorescens* group [1, 2] provide a compelling example of ecological and bacterial lifestyle diversity reflected by the vast range of environmental habitats they occupy. This group encloses plant-beneficial symbionts, environmental saprophytes and clinical strains of opportunistic human pathogens [3–5]. Within the *P. fluorescens* group, root-colonizing pseudomonads are well known for their ability to promote plant growth and to protect plants against soilborne pathogens through a set of diverse and functionally complementary mechanisms. The capacity to suppress fungal diseases has largely been attributed to the production of secondary metabolites with cytotoxic and antimicrobial activity, in particular 2,4-diacetylphloroglucinol (DAPG), phenazines, pyoluteorin, pyrrolnitrin, hydrogen cyanide, and lipopeptides [4, 6].

**Fig. 1:**
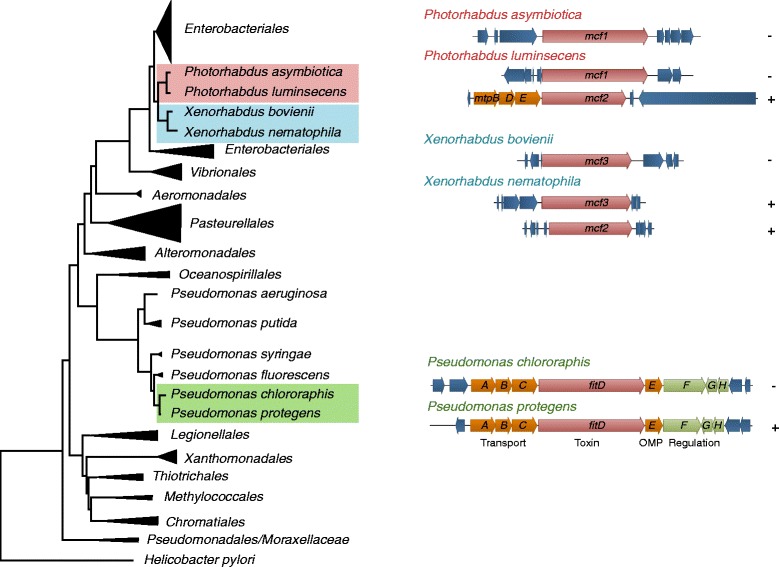
Organization of the Fit gene cluster and their homologues (*mcf*). In *P. protegens* and *P. chlororaphis* the *fit* gene cluster (*fitA-H*) is composed of eight ORFs encoding the insecticidal toxin (red arrow), predicted components of a toxin secretion system (orange arrows) including an outer membrane protein (OMP), and regulators of toxin production (green arrows). The orientation of the toxin gene within the genome is indicated by + for the leading strand and by – for lagging the strand. Transporter homologues (*mtpBDE*) are only found in *P. luminescens* adjacent to *mcf2*, a truncated variant of *mcf1. X. bovienii* SS2004 and *P. asymbiotica* ATTC 43949 harbor only one *mcf* variant. Blue arrows indicate flanking genes, which share no homology among the strains represented. The phylogenetic relationship of *P. protegens*, *P. chlororaphis*, *Photorhabdus* spp. and *Xenorhabdus* spp. with representative orders/families and groups of γ-proteobacteria is based on concatenated RecA, RpoB and RpoD protein sequences

Extensive knowledge has been gathered over the last years on plant disease suppression and plant growth promotion. Surprisingly, it has become only recently apparent that specific strains of plant-associated pseudomonads are able to infect and kill insects [7–12]. These observations invoke that particular strains may function as insect pathogens and switch between insect hosts and the plant environment. Insecticidal activity in environmental pseudomonads was, with the exception of *Pseudomonas entomophila,* a pathogen of *Drosophila* [13–15], so far only rarely demonstrated. Initially, an insect toxin was discovered *in silico* when the genome of *Pseudomonas protegens* Pf-5 (previously called *P. fluorescens* Pf-5) became available [16]. Subsequent molecular and mutational characterization revealed that oral and injectable insecticidal activity is linked to the Fit (*P*. *fluorescens* insecticidal toxin) gene, which was described and characterized for the first time in *P. protegens* strains CHA0 and Pf-5 [7, 12]. Injection of Fit expressing *E. coli* is sufficient to induce strong melanization and rapid death of the tobacco hornworm *Manduca sexta* and larvae of the greater wax moth *Galleria mellonella* [7]. Fit toxin knock-out mutants of CHA0 have attenuated virulence, both when injected into *G. mellonella* or fed to African cotton leafworm *Spodoptera littoralis* [7, 12]. The Fit gene cluster consists of eight genes (*fitABCDEFGH*) with functions in toxin export, insect toxicity and regulation (Fig. 1). The Fit insect toxin gene *fitD* is flanked upstream by *fitABC* and downstream by *fitE* encoding components of a type I secretion system. The products of the *fitFGH* genes regulate toxin production [7, 8, 10, 11]. FitF is a sensor histidine kinase – response regulator hybrid, detecting the insect environment and activating insecticidal toxin expression via FitH and FitG [11].

Genome sequencing revealed that certain strains of *Pseudomonas chlororaphis* also harbour the complete *fit* gene cluster (Fig. 1) [17]. Contribution of the Fit toxin to the oral insecticidal activity has been demonstrated for *P. chlororaphis* PCL1391 against *S. littoralis* [12]. The Fit insect toxin shares 73 % identity with the makes caterpillars floppy insecticidal toxin Mcf1 and 67 % with Mcf2, both produced by *Photorhabdus luminescens*, a bacterial symbiont of entomopathogenic nematodes [7, 18, 20]. Mcf-like toxins are also found in *Photorhabdus asymbiotica*, *Xenorhabdus nemtophila* and *Xenorhabdus bovienii* (Fig. 1) [18–21]. The Mcf1 toxin causes rapid disruption of the insect midgut epithelium and hemocytes triggered by a BH-3-like apoptosis control domain [18, 22]. Injection of purified Mcf1 in *Drosophila* embryos leads to a freezing phenotype of hemocytes, due to a rearrangement of the actin cytoskeleton [23]. While Mcf toxins are essentially studied in the *Photorhabdus* lineage the evolutionary basis for the homology between Fit and Mcf toxins has remained unclear.

Here, we conducted comparative sequence analysis in combination with virulence assays to yield a better understanding of insect pathogenicity in plant-associated pseudomonads. In order to study Fit/Mcf toxin evolution we have sequenced seven *Pseudomonas* genomes to retrieve the entire gene cluster. We analyzed evolutionary footprints of the *Pseudomonas* Fit gene cluster and the related Mcf genes of the insect pathogenic *Photorhabdus* and *Xenorhabdus* bacteria and found patterns of recent horizontal transfer.

This study suggests that the Fit toxin is restricted to a particular group of plant-colonizing pseudomonads consisting of *P. protegens* and *P. chlororaphis*. We show that the presence of the *fit* toxin gene strongly correlates with high insect toxicity and thus is a suitable molecular marker for potent insecticidal activity in fluorescent pseudomonads. Absence of the Fit toxin gene in closely related pseudomonads and the genomic context suggest that *fit* genes have evolved in part via exchange of genetic material from phylogenetically distantly related bacteria. The acquisition of the Fit toxin within pseudomonads may represent an ancient event in the evolution towards a distinct ecotype of insect-associated pseudomonads. Our analysis further indicates substantial rearrangements within *Photorhabdus/Xenorhabdus* lineage of these insecticidal toxins thereby extending and diversifying the existing toxin repertoire of these entomopathogens.

## Results and discussion

### Survey of diverse pseudomonads predicts *P. protegens* and *P. chlororaphis* as exclusive Fit producers within plant-colonizing pseudomonads

**Fig. 2:**
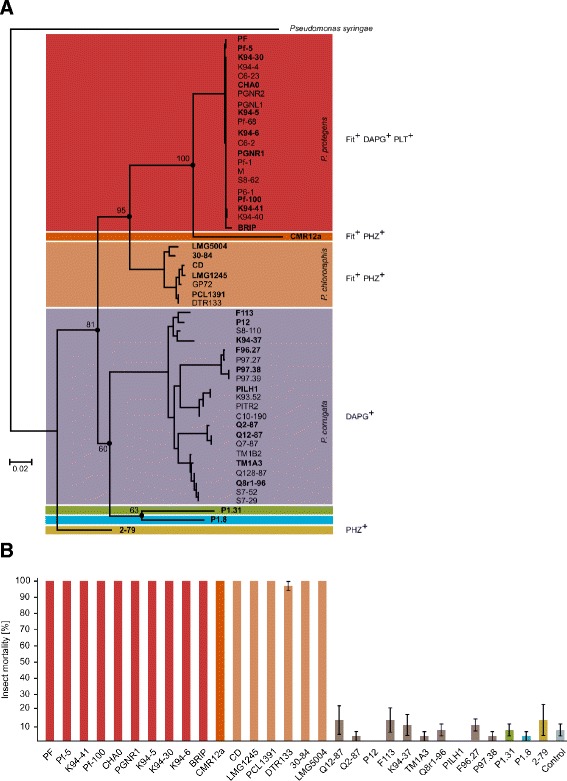
Insecticidal activity correlates with presence of the *fitD* gene. (**a**) The Phylogenetic relationship amongst 29 closely related plant-associated Fit^+^ and 24 Fit^−^ pseudomonads is based on the three concatenated housekeeping genes *recA*, *rpoB* and *rpoD* consisting of a total of 1469 nucleotide sites with *Pseudomonas syringae* as outgroup. Strains/subgroups harboring the Fit toxin gene and major antifungal compounds produced by the pseudomonads are indicated: DAPG, 2-4-diacetylphloroglucinol; PHZ, phenazines; and PLT, pyoluteorin. The evolutionary history was inferred using PhyML 3.0. Bootstrap values are based on 100 replicates and indicated for major nodes indicated by circles. Only the *P. protegens* and *P. chlororaphis* subgroups harbor the Fit toxin gene. The *P. corrugata* subgroup containing DAPG producers is indicated as defined by Mulet [1, 2]. (**b**) Insect mortality of *G. mellonella* larvae four days after injection of 4 × 10^4^ cells of 17 Fit producing and 13 non-producing *Pseudomonas* strains. Tested strains are shown in bold in (A). Each strain was tested on a total of 30 larvae (five replicate plates with six larvae per plate). Bars show average of insect mortality for each strain. Error bars show standard error of the mean. The experiment was repeated with similar results (Additional file 2: Table S2). Fit^+^ strains were significantly different from Fit^−^ based on Wilcoxon rank sum test grouped by Fit^+^ and Fit^−^ strains (*P* < 0.05)

We investigated the occurrence of Fit toxin production in plant-associated pseudomonads and tested whether insect toxin production is linked to specific ecological and molecular characteristics. We screened a large worldwide collection of *Pseudomonas* isolates (103) from soil and roots of different plant species using generic primers directed against the Fit toxin gene *fitD* (Additional file 1: Table S1). In addition to the root-associated isolates, we tested 15 strains representing the major phylogenetic groups within the genus *Pseudomonas* and strains isolated from different environments including invertebrates such as cyclops, earthworms or isopods for the presence of the *fitD* gene. The phylogenetic relationship of the investigated strains based on concatenated sequences of the three housekeeping genes *recA*, *rpoB* and *rpoD* is shown in Fig. 2a.

PCR amplification and sequencing showed the presence of the toxin in 29 strains (Fig. 2a, Additional file 1: Table S1). In addition, PCR results were verified using Southern blotting on a subset of isolates (data not shown). We detected the Fit toxin gene only in two phylogenetic subgroups within the *P. fluorescens* group (grouping according to [1, 2]). The first subgroup comprises fluorescent pseudomonads that produce both the antifungal metabolites DAPG and PLT [24, 25] including our model strain *P. protegens* CHA0 [26]. As the second group of Fit toxin gene carriers, we were able to identify members of the *P. chlororaphis* subgroup (Fig. 2a). We included in addition to *P. protegens* CHA0 and Pf-5 the sequenced strains *P. chlororaphis* GP72 [27], *Pseudomonas aureofaciens 30-84* [17] and *Pseudomonas* CMR12a (unpublished data) all harboring the Fit gene cluster. CMR12a is placed next to the group of DAPG and PLT producers, although CMR12a is phylogenetically clearly distinct from these strains (Fig. 2a) and does not produce the two antifungal compounds [28, 29].

### Insecticidal activity strongly correlates with the presence of the Fit toxin gene

We aimed to test if insecticidal activity is restricted to a particular group of pseudomonads and whether presence of the Fit toxin is predictive of insecticidal activity. To this end, the insecticidal activity of selected strains from phylogenetically and functionally diverse subgroups within the *P. fluorescens* group (Fig. 2a) was tested using a previously established assay with larvae of the greater wax moth *Galleria mellonella* [7]. Tested strains included representatives of different phylogenetic subgroups of DAPG-producers and some DAPG non-producing strains (Fig. 2a). Injection assays with *Galleria* larvae demonstrated that *Pseudomonas* strains harboring the *fitD* toxin gene display potent insecticidal activity while the naturally *fitD*-negative sister group fails to induce significant mortality (Fig. 2b). Since insecticidal activity is restricted to a particular group and substantial toxicity is retained in *fitD* knock-out mutants [7], insecticidal activity is likely driven by other shared traits. We have previously shown that the *fit* toxin gene significantly contributes to the insecticidal activity, but requires additional factors for full activity that are regulated by the GacS/GacA system [11, 12]. Nevertheless, the Fit toxin gene seems to be predictive of insecticidal activity in root-colonizing fluorescent pseudomonads.

### The Fit gene cluster is located in a dynamic genomic region

**Fig. 3:**
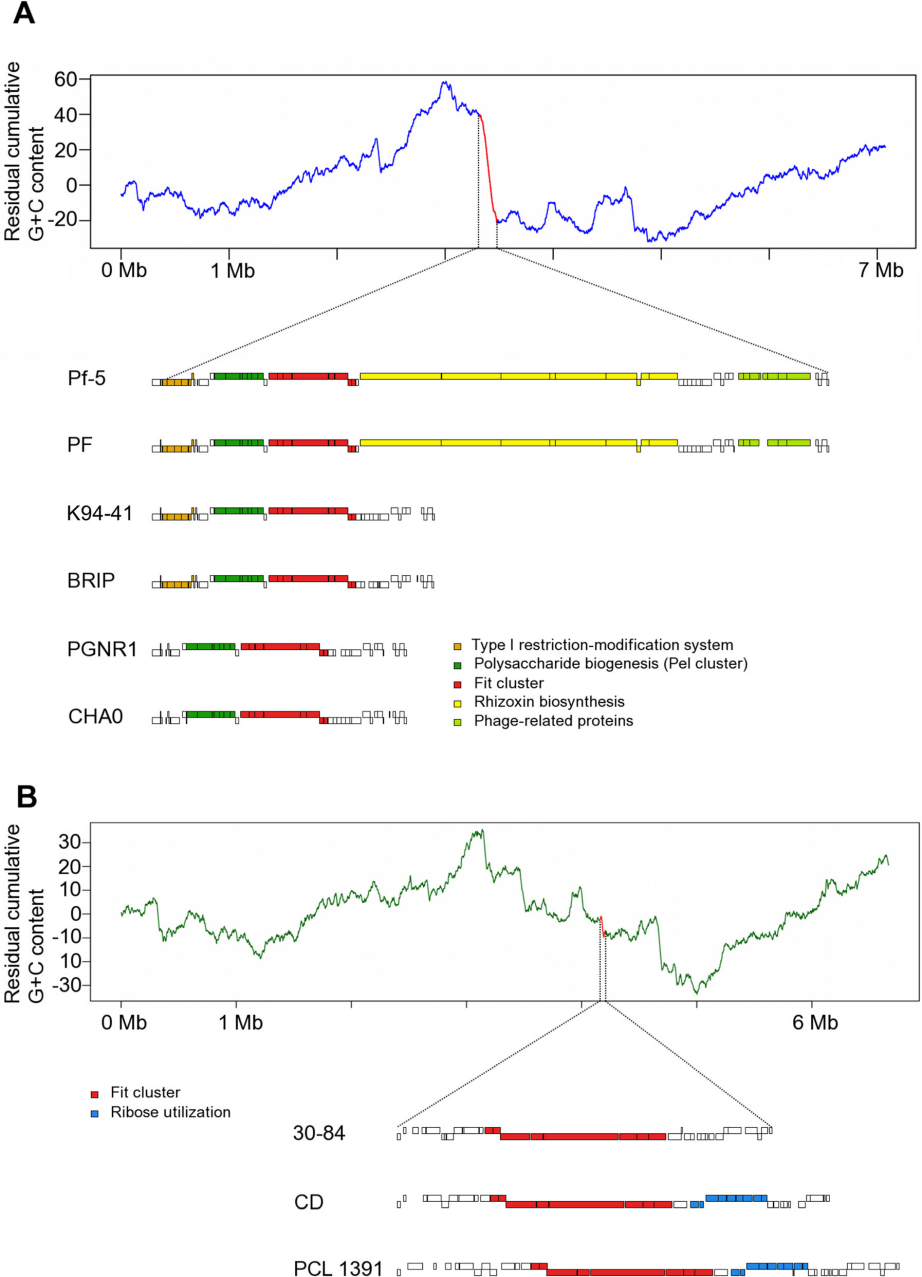
Comparison of the Fit cluster harbouring region. Within *P. protegens* (**a**) and *P. chlororaphis* (**b**) subgroups indicates a high degree of absence/presence polymorphism. The genomic region was defined based on residual cumulative G + C content analysis of *P. protegens* strain Pf-5 and *P. chlororaphis* strain 30-84, where steep slopes indicate local variations in G + C content indicative of foreign elements. Compared with Pf-5 and PF, the genomic region of strains K94.41, BRIP, PGNR1 and CHA0 is notably downsized

To characterize the Fit gene cluster among *Pseudomonas* spp. we generated high quality assemblies of seven toxin-producing strains (i.e., *P. protegens* CHA0, BRIP, PGNR1, K94.41, and PF and *P. chlororaphis* PCL1391 and CD of which) covering the entire Fit toxin cluster and flanking regions. The complete genome sequence of CHA0 has recently been published [30]. Sequences containing the *fi*t genes were aligned to the reference sequence of strains Pf-5 and 30-84 [16, 17]. All seven strains contain the complete *fit* toxin cluster (*fitABCDEFGH*). The Fit gene cluster in the genome of Pf-5 is embedded in a large genomic region, with features indicative of horizontal acquisition, such as phage-related proteins and unusual nucleotide composition (Fig. 3a) [17]. The *fit* cluster in *P. chlororaphis* strains 30-84 and O6 is located in a different part of the genome within a 24-28 gene insertion (Fig. 3b) [17]. We were able to define a 165-kb region of the Pf-5 genome flanked by steep residual G + C content clines (Fig. 3a). Abrupt changes in the residual cumulative G + C content curve may point to foreign genetic elements, such as horizontally acquired genes, phage-derived elements or other mobile elements [31]. The corresponding genomic region in strain PF is highly similar to that of Pf-5 and in both strains the region is delimited at the 5’-end by a type I restriction modification system and by phage-related proteins at the 3’-end. Interestingly, comparisons of the respective regions among the six *P. protegens* strains show a high level of insertion-deletion polymorphism. In particular, the type I restriction-modification system is present in strains Pf-5, PF, K94.41 and BRIP, but absent in PGNR1 and CHA0 (Fig. 3a). The gene cluster encoding the rhizoxin biosynthesis is unique to Pf-5 and PF as well as the phage-related proteins [32]. A polysaccharide synthesis gene cluster (*pel*) is common to all *P. protegens* strains and located upstream of the Fit cluster. In *Pseudomonas aeruginosa*, Pel is one of at least three secreted extracellular polysaccharides implicated in biofilm formation [33].

The genomic context of the *fit* genes in *P. protegens* differs from that in *P. chlororaphis* (Fig. 3b). Using the residual cumulative G + C content approach a region of 45 kb composed of 34 ORFs was defined in *P. chlororaphis* strain 30-84. Similar to *P. protegens*, there is substantial presence/absence polymorphism. In strains CD and PCL1391, adjacent to a gene encoding a putative membrane-associated transporter upstream of *fitA*, a nine-ORF gene cluster involved in ribose uptake and utilization is located, which was assumed to be unique to the *P. chlororaphis* subgroup of the *P. fluorescens* group [17].

### Fit gene cluster: a phylogenetic patchwork

**Fig. 4:**
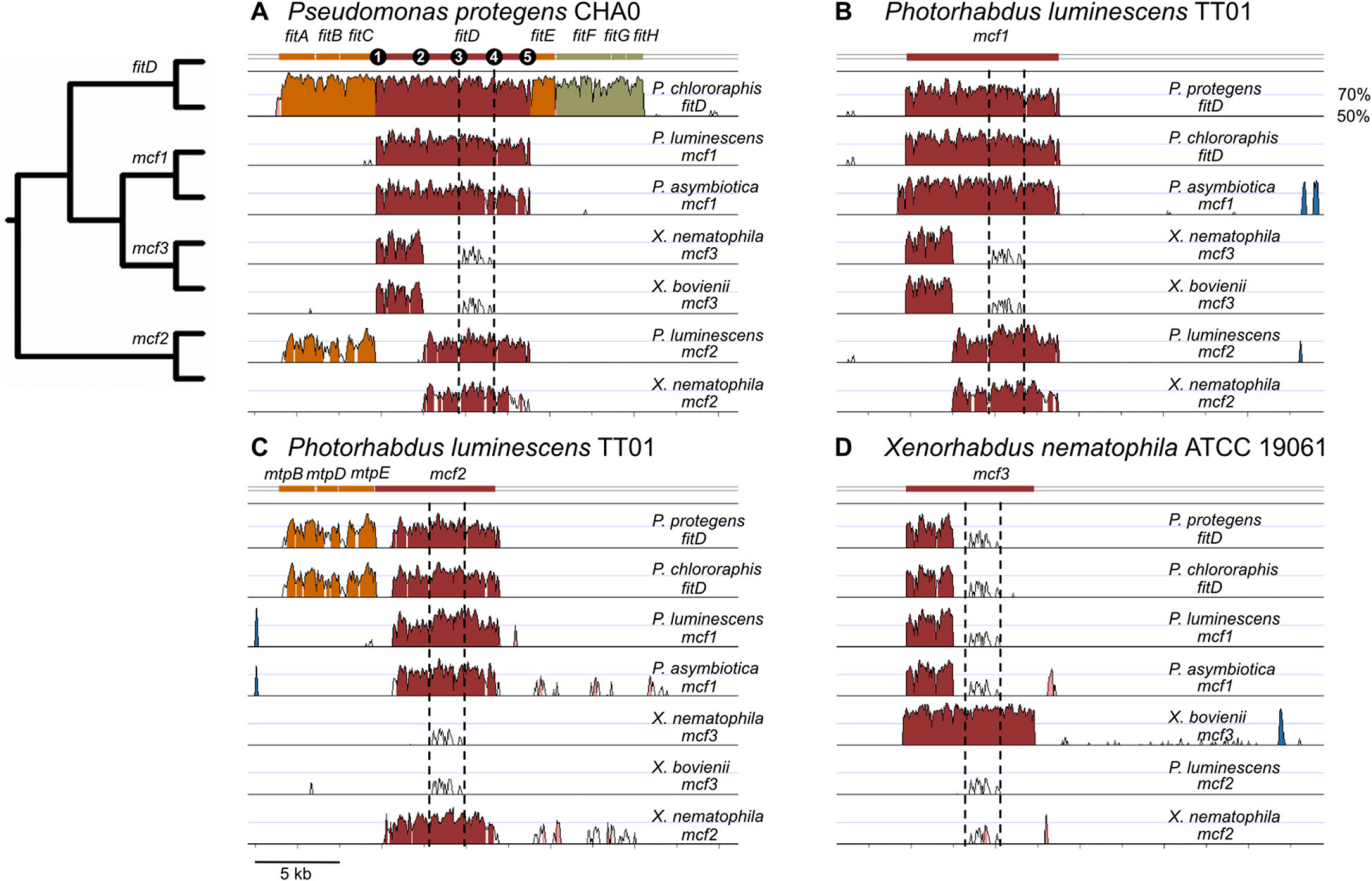
Similarity plot Fit and Mcf toxin encoding genomic regions in *Pseudomonas*, *Photorhabdus* and *Xenorhabdus* species. The alignment was conducted using LAGAN as implemented in mVISTA [59] with the respective reference sequence of (**a**) *P. protegens* CHA0 *fitD*, (**b**) *P. luminescens* TT01 *mcf1* (**c**) *Photorhabdus luminescens* TT01 *mcf2* and (**d**) *X. nematophila* ATCC 19061 *mcf*. The peaks and valleys graphs represent percent conservation between aligned sequences at a given coordinate on the reference sequence. Regions of high conservation (≥70 %) are colored according to the coding region of the reference sequences in dark red for toxin genes, in orange for transport genes and in green for regulation genes. Regions of high conservation not related to the cluster sequence are colored in pink. Regions colored in blue have high similarity to transposable elements and are only present adjunct to *mcf* genes in *Xenorhabdus* and *Photorhabdus*. Dotted lines mark the region encoding the TcdA/TcdB pore forming domain. Nucleotide key positions discussed in the text are indicated with numbers 1-5 in Fig. 1a for *fitD*: 1 = site 1; 2 = site 2707; 3 = site 4849; 4 = site 6831; 5 = site 9015. The plot shown of each alignment ranges between 50 % and 100 % identity calculated on a 100 bp window

Since only strains of *P. protegens* and *P. chlororaphis* were identified as carriers of the Fit toxin, we were interested in the evolutionary origin of this gene cluster. High similarities between protein sequences in distantly related species, patchy distribution or phylogenetic incongruence indicate potential horizontal transmission of a gene [34]. BLASTP searches, revealed a very distinct phylogenetic distribution of Fit components. Consistent with horizontal transmission, most of the Fit components (except FitE and FitG) have significant best hits outside the *Pseudomonas* (Additional file 2: Table S2). Initial sequence analysis of the Fit toxin from *P. protegens* strain CHA0 revealed 73 % sequence identity over the entire protein to the insecticidal protein Mcf1 of *P. luminescens* strain TT01 [7]. Mcf2 of TT01 shares 67 % identity with FitD (Additional file 3: Figure S1). Mcf1 is also present in *P. asymbiotica* ATCC 4394, but this bacterium appears to have lost Mcf2 [20]. Similarly, *X. bovienii* SS-2004 and *X. nematophila* ATCC 19061, both sister species of *Photorhabdus*, carry an Mcf variant, which we call here Mcf3 with 69 %, respectively, 65 % overall identity to FitD and highest identity (79 %, respectively 76 %) in a 900 amino acid N-terminal overlap. Mcf3 is also present in *Photorhabdus temperata* [35]. Within the genus *Xenorhabdus* Mcf2 is only found in *X. nematophila* ATCC 19061 with an overall identity of 64 % to FitD. Mcf-like proteins are also found in other γ-Proteobacteria including *Vibrio* and *Providencia* spp. Distantly related Fit-like genes (27-28 % identity) [7, 17], (Additional file 2: Table S2) with a predicted TcdA/TcdB pore-forming domain can also be found in *P. brassicacearum* and diverse Fit-negative *P. fluorescens* strains, but none of the representative strains of this group tested in our virulence tests (i.e., P12, Q8r1-96, Q2-87, Q12-87, belonging to the *P. corrugata* subgroup) caused significant insect mortality (Fig. 2b). Interestingly, FitA, FitB and FitC with predicted function in toxin secretion [7] revealed highest similarities (69 %, 65 % and 73 % amino acid identity) with the RTX toxin transporter encoded by the genomic region adjacent to the Mcf2 insect toxin gene of *P. luminescens* (Additional file 2: Table S2, Fig. 4). Similar genes, however, are absent in proximity of *mcf1* and *mcf3* as well as of *mcf2* of *Xenorhabdus* (Figs. 1 and 4). The only *fit* components showing closest identity to the genus *Pseudomonas* are FitE (62 % identity to *P. brassicacearum*) and the regulatory protein FitG (45 % identity to *P. fluorescens* and *Serratia* sp.). The other proteins involved in regulation of toxin expression (FitF and FitH) show closest identity to *Dechloromonas aromatica* (41 %) and *Vibrio* sp. (54 %), respectively. The three regulatory proteins FitF, FitG and FitH are absent in *Photorhabdus*/*Xenorhabdus*, suggesting a *Pseudomonas*-specific regulation of Fit. However, we cannot rule out an earlier existence of the whole cluster also in *Photorhabdus*/*Xenorhabdus* but that transport and regulatory genes were subsequently lost. Mcf2 in *Photorhabudus luminescens*, for example is still flanked by the three transport genes.

The patchy phylogenetic distribution of Fit/Mcf toxins and the absence of the toxin in closely related *Pseudomonas* species e. g members of the *P. corrugata* subgroup strongly suggest that the Fit cluster evolved in part via horizontal acquisition, followed by functional integration of vertically transmitted genes, making up a unique virulence cluster within the subclade of *P. chlororaphis* and *P. protegens*.

### Mosaic composition of Fit/Mcf toxin variants

Aligning the genomic regions of FitD and Mcf variants encoding sequences reveals a mosaic like structure of toxin-encoding parts and associated components. The *fitD*-encoding region of *P. protegens* and *P. chlororaphis* shows extraordinarily high similarity over the entire gene (69-75 %) to *mcf1* of *P. luminescens* and *P. asymbiotica*. However, immediately adjacent flanking regions drop below the threshold of alignable sequences set at 50 % similarity in a 100 bp window (Fig. 4). Short stretches sharing over 70 % nucleotide similarity outside the designated toxin encoding regions indicated by sharp peaks in the similarity plots among *P. luminescens*, *P. asymbiotica* as well as between *P. luminescens* genes *mcf1* and *mcf2* include fragments with predicted association to transposable elements (Fig. 4).

Within the *Photorhabdus*/*Xenorhabdus* lineage three variants of Mcf toxins can be determined based on their sequential make up: Mcf1, Mcf2 and Mcf3. While insect toxicity has been demonstrated at several levels for Mcf1 and Mcf2 from *Photorhabdus* [18, 19, 23], and FitD from *Pseudomonas* 2013 [7, 12], the functionality of *Xenorhabdus* Mcf toxins remains to be tested, in particular of Mcf3 the most distinct toxin variant discussed in this paper.

**Fig. 5:**
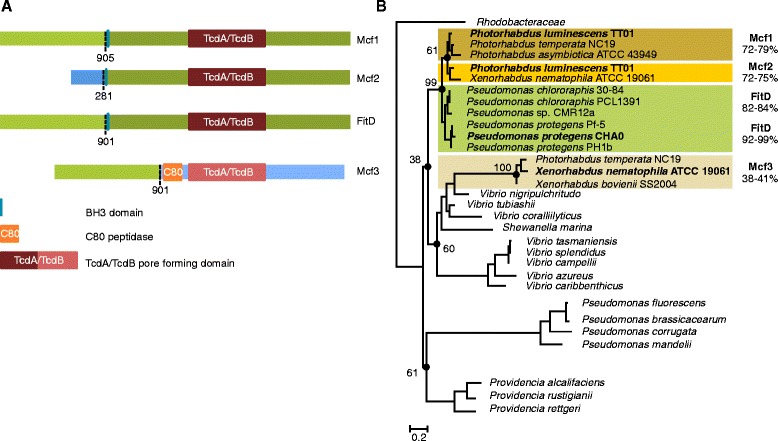
Representation of Fit and Mcf toxin variants. (**a**) Segments of same color represent highly identical regions (>60 % identity) between the different variants. Strains representing the toxin variants are indicated in bold in Fig. 1b. C80 peptidase is only present in Mcf3. TcdA/TcdB indicates the pore-forming domain present in all variants, but most likely of different origin (Fit/Mcf1/Mcf2 vs. Mcf3). The BH3 domain present in FitD, Mcf1 and Mcf2 has been attributed a functional role in cell death induction [22]. (**b**) Phylogenetic relationship of the bacterial TcdA/TcdB pore-forming domain present in all Fit and Mcf protein variants. The TcdA/TcdB domain from *Rhodobacteraceae* was designated as outgroup*.* The tree is based on 312 amino acids after removal of segments by Gblocks [54] and inferred using PhyML 3.0 with the HKY85 substituion model and default settings [55]. Bootstrap values are based on 100 replicates and given for relevant nodes indicated by circles. The range of amino acid identity of the TcdA/TcdB domain compared to the FitD reference sequence of CHA0 is given next to the colored boxes delimiting the different Mcf variants

The VISTA alignment presented in Fig. 4 shows the mosaic-like structure of Mcf variants. While *fitD* and *mcf1* share high homology over the entire nucleotide sequence, *mcf3* in the *Xenorhabdus* lineage and also present in *P. temperata* shares only the 5′-end (Position 1-2, Fig. 4) with *fitD*/*mcf1* (Figs. 4 and 5a). Including *mcf2* in the alignment, it becomes apparent, that it is exactly this stretch at the *mcf3* 5’ -end with high homology to *fitD*/*mcf1*, which is entirely missing in *mcf2* (Figs. 4 and 5). BLAST searches revealed that the sequence towards the 3′-end (Position 2-5, Fig. 4) of the *Xenorhabdus mcf3* shows for most of this stretch (position 2-3 and position 4-5, Fig. 4) no similarity to the other *mcf/fitD* variants and did not allow to identify a putative origin. The rapid drop from >70 % similarity to 40 % between position 1 and 2 (Fig. 4) coincide with a breakpoint indicated by SBP (Single Breakpoint Recombination) analysis. In the middle of this stretch there is a part (position 3-4, Fig. 4) which again shows a low similarity (50-70 % at the nucleotide level, Fig. 4) to the TcdA/TcdB pore forming domain of *fit*, *mcf1* and *mcf2.* Thus, all investigated toxin variants encode a TcdA/TcdB pore-forming domain in this region, however, based on amino acid and nucleotide comparison, it seems that these pore-forming domains originate from at least two different ancestors (Fig. 4). A phylogenetic tree conducted on the TcdA/TcdB pore forming domain indeed suggests a monophyletic origin for the pore-forming domain of FitD/Mcf1 and Mcf2, but a different origin for that of Mcf3 found in *Xenorhabdus* and *P. temperata* (Fig. 5b).

The mosaic composition of the different *fit*/*mcf* variants, the presence of transposable elements and the patchy phylogenetic distribution of the toxin could indicate a highly mobile nature of this gene. An unusual GC content of a gene/segment, is a further indication of horizontal acquisition, assuming that donor and recipient have a sufficiently high degree of base composition differences [36, 37]. While the average GC content of *P. luminescens* TT01 (43.9 %) and *X. nematophila* ATCC 19061 (45.3 %) genomes is low, *Pseudomonas* spp. have high overall GC content (*P. protegens* Pf-5: 64.0 %; *P. chlororaphis* 30-84: 63.8 %). Comparing the GC content of *fitD* and *mcf1* to the respective average of all genes within the same strain, the *mcf1* gene in *P. luminescens* displays an unusual high GC content (56.2 % vs. 43.9 %), whereas the *fitD* gene in *Pseudomonas* is within the range of the average GC content of the genome (e.g., Pf-5: 65.2 % vs. 64.0 %) (Table 1). In addition, only 0.3 % of *P. luminescens* genes display an equal or higher GC content than *mcf1* and *rtxD* and *rtxB* (homologous to *fitA* and *fitB* in *P. luminescens* adjunct to *mcf2*) (data not shown). Interestingly, all investigated *mcf* variants of *Xenorhabdus* spp. do not show such obvious abnormality (50 % vs. 45 %) (Table 1).

**Table 1:** GC content of *fitD* and *mcf* genes

Strain	Gene	Position	Strand	Length (bp)	GC content (%)	GC content genome (%)
*Pseudomons protegens*						
Pf-5	*fitD*	3350746..3359757	+	9012	65.2	64.0
CHA0	*fitD*	3362990..3371995	+	9006	65.1	63.4
	*fitA*	3357171..3359312	+	2142	65.4	
	*fitB*	3359309..3360697	+	1389	65.7	
	*fitC*	3360700..3362859	+	2160	66.9	
*Pseudomonas chlororaphis*						
3084	*fitD*	4176228..4185206	+	8979	66.1	62.9
*Photorhabdus luminescens*						
TTO1	*mcf1*	4832190..4841195	-	8994	56.2	43.9
	*mcf2*	3670273..3677427	+	7155	52.0	
	*Plu*3125^a^	3664486..3666606	+	2121	54.8	
	*Plu3126* ^a^	3666606..3667994	+	1389	55.8	
	*Plu3127* ^a^	3667994..3670153	+	2160	57.3	
*Photorhabdus asymbiotica*						
ATCC 43949	*mcf1*	3962994..3971975	+	5982	54.4	42.2
*Xenorhabdus nematophila*						
ATCC 19061	*mcf*	2205090..2212682	+	7593	50.7	45.3
	*mcf2*	1917776..1924951	+	7176	50.2	
*Xenorhabdus bovienii*						
SS-2004	*mcf*	2380592..2388193	-	7602	50.6	45.0

^a^Encode predicted transporters and are homologous to *fitA*, *fitB* and *fitC* respectively

The unusual nucleotide composition of *mcf1* and *mcf2* in *Photorhabdus* contradicts the hypothesis that pseudomonads acquired the insect toxin from entomopathogenic *Photorhabdus*. However, horizontal acquisition of *mcf* in *Photorhabdus* from an unknown ancestral vector is very likely. In P*. chlororaphis* and *P. protegens* the nucleotide composition of the *fit* genes does not differ from the average of the whole genome (Table 1). Therefore, one might speculate that pseudomonads have acquired the toxin earlier than *Photorhabdus* and the *fit* codon usage has already adapted to the *Pseudomonas* background or that pseudomonads have acquired the toxin from a bacterium displaying a similar nucleotide composition.

*mcf* toxin genes are not only shuffled around in bacteria. A recent study by Ambrose [38] indicates that a *mcf*-like gene of the fungal grass endosymbiont *Epichloë poae*, which is sufficient to confer a lethal phenotype when expressed in *E. coli* cells and injected into the black cutworms *Agrotis ipsilon,* has derived from a single lineage-specific horizontal transfer of bacterial origin [38].

## Conclusions

The plant environment was assumed to be the dominant niche of *P. fluorescens* group bacteria, but it becomes apparent that some members, notably *P. protegens* and *P. chlororaphis*, which harbor the Fit insect toxin, are capable of colonizing and killing insects [7–12, 17]. The present comparative analysis study provides a better understanding of the processes driving the evolution of insect pathogenicity in environmental pseudomonads. The Fit virulence cassette seems to be ubiquitous for *P. protegens* and *P. chlororaphis* and is encoded in dynamic portions of the *P. protegens* and *P. chlororaphis* genomes with substantial absence/presence polymorphism, phage-related genes and an unusual base composition, while in the *Photorhabuds/Xenorhabdus* lineage transposable elements are located in proximity of the Mcf gene. It would therefore appear that evolutionary processes including the acquisition of insecticidal elements, sequence rearrangements (as demonstrated in this study) and protein adaptation through domain shuffling (as demonstrated by Kupferschmied [11]) allowed plant-associated pseudomonads to adapt to a new ecological niche. In line with the genomic arguments of horizontal acquisition are the patchy distributed *fit* components that share highest homology with bacteria outside the *Pseudomonadaceae* family. Our data show that a specific group of plant-colonizing pseudomonads have evolved a unique virulence gene cluster through diverse evolutionary processes, which contributed to extend their existing repertoire of antifungal and antipredator activities with insecticidal activity. Frequent mobilization and recombination is possibly favored by the common niche of insect hosts shared between these particular *Pseudomonas*, *Photorhabdus* and *Xenorhabdus* bacteria, and may provide a selective advantage by the diversification of the toxin gene repertoire.

## Methods

### Bacterial strains

Bacterial strains used in the present study are summarized in Additional file 1: Table S1. For the screening, we relied on a worldwide strain collection of *Pseudomonas* spp. isolated from the rhizosphere, roots and leaves of various plant species [24, 25, 39–41]. Strains belonging to the genus *Photorhabdus* and *Xenorhabdus* were originally isolated from entomopathogenic nematodes (*Steinernema* and *Heterorhabditis* species) sampled from diverse soils in Switzerland using *Galleria mellonella* larvae as baits [42]. Bacteria used in this study were cultured on King’s medium B (KMB) agar plates, or in lysogeny broth (LB) at 27 °C [43–45]. Additional strains from environmental samples were isolated by plating serial dilutions on KMB supplemented with antibiotics at the following concentrations: chloramphenicol 13 μg ml^−1^, ampicillin 40 μg ml^−1^ and cycloheximide 100 μg ml^−1^. For single gene amplification, DNA was obtained from overnight LB cultures diluted 1:500 with sterile distilled H_2_O and incubated for 10 min at 96 °C to lyze bacterial cells.

### Insect toxicity assay

Washed bacterial cells from overnight cultures in (LB) were suspended in 0.9 % sterile NaCl solution and adjusted to an OD_600_ = 0.01. Aliquots of 5 μl, corresponding to an injection dose of 4 x 10^4^ cells, were injected into the haemolymph of ultimate-instar *G. mellonella* larvae (Hebeisen Fishing, Zürich, Switzerland) using a Hamilton microsyringe with a 26-gauge needle [7]. Sterile NaCl solution served as control. Treated larvae were incubated in Greiner six-well plates at room temperature and scored as live or dead regularly over four days. For each bacterial strain, five replicate plates with six larvae per plate were prepared. The experiment was repeated twice with similar results. Mortality was defined as the inablity of larvae to react to poking. Significance between Fit^+^ and Fit^−^ pseudomonads was assessed based on Wilcoxon rank sum test (P ≤ 0.05). For data analyses, R version 3.1.1 was used [46].

### Taxon determination

For the taxon determination of uncharacterized strains, a 455-bp 16 s rDNA fragment was amplified and sequenced using the universal primers f933 and r1387 [47]. For strains belonging to the genus *Pseudomonas* three housekeeping genes were used in addition, amplified and sequenced with primers recAf1, recAr1 for *recA* (537 bp), rpoBf1, rpoBr1 for *rpoB* (508 bp) and rpoDf1, rpoDr1 for *rpoD* (695 bp) [24]. PCR reactions were conducted according to the standard protocol for use of Dream TAQ Polymerase (Fermentas GmbH, St. Leon-Rot, Germany). PCR products were electrophoreticaly separated on 1 % agarose gels and purified using the Nucleo-Fast PCR purification kit (Macherey-Nagel). Sequencing was carried out using the BigDye® Terminator v3.1 Cycle Sequencing Kit (Life Technologies Cooperation, Carlsbas, USA). Sequencing products were purified on Sephadex G-50 followed by capillary elctrophoresis separation using an ABI Hitachi 3130xl Prism Genetic Analyzer (Applied Biosystems). The obtained sequences were blasted against public available genomic sequences on the NCBI website.

### Sequencing of *fit* gene cluster and *fitD*/*mcf1* fragments

Pairs of primers for the amplification of *fitD*/*mcf1* genes were designed based on the *fitD* sequences of *P. protegens* strains CHA0 and Pf-5. Primer specificity was tested *in silico* using BLASTN against assemblies of whole genome shotgun sequences from the NCBI website. Primer pairs are fit1f 5’-TGGCTTTTATGTCCAAGGAC-3’, fit1r 5’-TGGTTGGCGAAGTACTGCTC-3’ (position 2-962) and fit2f 5’-CTGACCACGTTCGACGCCGAGCAATG-3’, -fit2r 5’-TAACGTCCCACCGCCTTGGCATCTTCG-3’ (position 4828-5702) and allowed amplifiaction of *fitD, mcf3* and *mcf1*, but not *mcf2*. The fit1f/fit1r primer pair was tested on a collection of *Pseudomonas, Photorhabdus* and *Xenorhabdus* spp. listed in Additional file 1: Table S1. Amplification with primers fit1f and fit1r yielded one single amplicon ranging from 914 to 980 bp for *P. protegens*, *P. chlororaphis, Photorhabdus* and *Xenorhabdus* strains carrying the *fitD*, *mcf3* or *mcf1* insect toxin genes, respectively. For *P. protegens* and *P. chlororaphis,* a second *fitD* fragment was amplified and sequenced with primer pair fit2f/fit2r resulting in one single amplicon of 875 bp. PCR reactions and sequencing of the two fragments were conducted as described above for housekeeping genes.

Illumina sequencing was applied to retrieve the entire *fit* cluster and flanking genes of *P. protegens* strains PF, K94.41, BRIP, PGNR1, and CHA0 and *P. chlororaphis* strains PCL1391 and CD. Genomic DNA was extracted from 10 ml LB overnight culture grown from a single colony using the DNeasy extraction kit (Qiagen). Sequence data consisted of 90-bp paired-end Illumina reads carried out on a 500-bp library. The short reads were assembled using SOAPdenovo version 1.05 [48]. Contigs that harbor the *fit* genes were identified by BLASTN searches and annoted on the RAST server [49]. The sequence obtained for CHA0 is in accordance with the recently published genome of CHA0 (NCBI accession no. CP003190, [30].

Vista alignments shown in Fig. 4 were performed using mVISTA [50, 51] with LAGAN as alignment algorithm on 100 bp window. The genomic region encoding either the Fit toxin for *Pseudomonas* species or the homologous Mcf toxin in *Photorhabdus* and *Xenorhabdus* (NC005126, NC012962, NC014228, NC013892) species were extracted using BioEdit (http://www.mbio.ncsu.edu/BioEdit/bioedit.html) 25 kb downwards and 25 kb upwards from the starting codon of the toxin encoding gene (*fit/mcf*) and oriented according to the transcription direction of *fitD* from *P. protegens* CHA0.

### Phylogenetic analysis

Public available gene sequences for housekeeping genes of previously characterized *Pseudomonas* and other γ-proteobacterial strains included in the phylogentic analyses were retrieved from GenBank (http://ncbi.nlm.nih.gov/genbank) and added to our dataset. For the phylogenetic analysis shown in Fig. 2 sequences of the three housekeeping genes (*recA*, *rpoB*, *rpoD)* were concatenated into a single combined dataset using BioEdit (http://www.mbio.ncsu.edu/BioEdit/bioedit.html). Sequence data sets were all aligned using MUSCLE [52] implemeted in MEGA5 [53] and alignment gaps and poorly aligned segements were removed with Gblocks [54] resulting in data sets of 1469 bp. Tree topologies based on nucleotide sequences (Fig. 2) were inferred using PhyML 3.0 with HKY85 substituion model and default settings [55]. The node supports were evaluated based on 100 bootstrap replicates.

The tree in Fig. 1 was inferred on concatenated whole protein sequences of RecA, RpoB and RpoD using the LG amino acid replacment matrix [56]. For the TcdA/tcdB phylogeny shown in Fig. 5, a BLASTP search was performed based on the amino acid domain sequence of *P. luminescens* of TT01 and the tree inferred as described above for protein sequences. The presence of the TcdA/TcdB pore-forming domain in this region was confirmed by SMART (http://smart.embl-heidelberg.de).

Single Breakpoint Recombination analysis on *mcf/fit* sequences were performed on the Datamonkey webserver (http://www.datamonkey.org).

### *In silico* detection of Fit components

The amino acid sequences of *fit* genes of *P. protegens* CHA0 [7] served as query for BLASTP searches against completed bacterial genomic sequences. BLAST searches are summarized in Additional file 3: Figure S1.

### Residual cummulative GC content

GC content for *fit* genes was calculated using the seqinr package implented in R [57]. Identification of the genomic region carrying the *fit* cluster was defined on local variations of G + C content of the *P. protegens* Pf-5 and *P. chlororaphis* 30-84 genomes. GC content of genomes as listed in Table 1 are retrieved from the NCBI genome database. The residual cumulative GC content analyses were conducted according to a GC profile approach [31] described previously by [58]. First, the G + C content is calculated in a 1-kb sliding window with 20-bp steps before the residual cumulative G + C content is presented as bi-dimensional graph on which chromosome positions on the horizontal axis are plotted versus the residues on the vertical axis. A DNA stretch enriched or depleted in G’s and C’s is indicated by a steep slope on the graphs in Fig. 3.

### Availability of supporting data

Sequence data supporting the results of this article are available in LabArchives (DOI:10.6070/H47M05X, http://dx.doi.org/10.6070/H47M05X0)

## Additional files

Additional file 1: Table S1. Bacterial strains used in this study [60–106]. Additional file 2: Table S2. BLASTp analysis of *P. protegens* CHA0 Fit components against completed bacterial whole genome sequences. Additional file 3: Figure S1. Insecticidal activity correlates with presence of the *fitD* gene.
