# Supplementary material for: Evolutionary patchwork of an insecticidal toxin shared between plant-associated pseudomonads and the insect pathogens Photorhabdus and Xenorhabdus
Source: BMC Genomics. 2015 Aug 16;16(1):609. doi: 10.1186/s12864-015-1763-2 (PMC4542124; doi:10.1186/s12864-015-1763-2)
Supplement: Additional file 1: Table S1. — Bacterial strains used in this study [60–106]. [file 12864_2015_1763_MOESM1_ESM.pdf]

Table S1 Bacterial strains used in this study.

Strain	Origin[a]	Fit /Mcf	Reference
Azospirillum brasilense			
UAP 154	Maize	-	[60]
CNF 535	Unknown	-	[61]
Azospirillum lipoferum			
Crt1	Maize	-	[62]
Bacillus mycoides			
A23	Unknown	-	[63]
Burkholderia spp.			
J2502	Unknown	-	[63]
Cupriavidus necator			
JMP134	Unknown	-	[64]
Erwinia amylovora			
CFBP 1430	Hawthorn	-	[65]
Erwinia carotovora			
ATTn10	Unknown	-	[66]
EC852	Unknown	-	[67]
Escherichia coli			
K12	Laboratory strain	-	[68]
Photorhabdus asymbiotica			
ATCC 43949	Human	+	[20]
Photorhabdus luminescens			
2	*Heterorhabditis* (Swiss soil)	+	This study
3	*Heterorhabditis* (Swiss soil)	+	This study
I	*Heterorhabditis* (Swiss soil)	+	This study
TT01	*Heterorhabditis bacteriophora*	+	[69]
Photorhabdus temperata			
1	*Heterorhabditis* (Swiss soil)	+	This study
7	*Heterorhabditis* (Swiss soil)	+	This study
9	*Heterorhabditis* (Swiss soil)	+	This study
10	*Heterorhabditis* (Swiss soil)	+	This study
17	*Heterorhabditis* (Swiss soil)	+	This study
Pseudomonas aeruginosa			
PAO1	Human	-	[70]
Pseudomonas caricapapaye			
LMG2152		-	LMG collection
Pseudomonas chlororaphis			
30-84	Soil	+	[71]
DTR 133	Soil	+	[72]
GP72	Green pepper	+	[27]
LMG 5004	Contaminated plate	+	[63]
LMG 1245	River Clay	+	[63]
CD	Cyclops (water)	+	This study
PCL 1391	Tomato	+	[73]
Pseudomonas corrugata			
LMG2172	Tomato	-	[74]
Pseudomonas fluorescens			
2-79	Wheat	-	[75]
C*1A1	Cucumber	-	[25]
C6-11	Tobacco	-	[39]
C6-16	Tobacco	-	[39]
C10-180	Tobacco	-	This study
C10-181	Tobacco	-	[39]
C10-186	Tobacco	-	[40]
C10-190	Tobacco	-	[40]
C10-197	Tobacco	-	[39]
C10-204	Tobacco	-	[39]
C10-205	Tobacco	-	[39]

CM1'A2	Cucumber	-	[76]
F113	Sugar beet	-	[77]
K92-1	Cucumber	-	This study
K92-5	Tomato	-	This study
K92-6	Tomato	-	This study
K92-8	Tomato	-	This study
K92-9	Tomato	-	This study
K92-11	Cucumber	-	This study
K92-12	Cucumber	-	This study
K92-14	Cucumber	-	[41]
K92-48	Cotton	-	[41]
K92-53	Cotton	-	[41]
K92-59	Cucumber	-	[41]
K93-2	Tobacco	-	[41]
K93-3	Tobacco	-	[41]
K93-7	Cucumber	-	[41]
K93-8	Cucumber	-	[41]
K93-9	Cucumber	-	[41]
K93-39	Wheat	-	[41]
K93-43	Wheat	-	This study
K93-48	Wheat	-	[78]
K93-52	Tomato	-	[41]
K93-53	Tomato	-	[78]
K94-18	Tomato	-	[41]
K94-31	Cucumber	-	[41]
K94-37	Cucumber	-	[41]
K95-7	Cucumber	-	[78]
KD	Wheat	-	[78]
LMG1794	Water	-	LMG collection
P1.8	Earthworm	-	This study
P1.31	Woodlouse (dead)	-	This study
P3	Barley	-	[79]
P12	Tobacco	-	[25]
P96.25	Wheat	-	[41]
P97.26	Tomato	-	[41]
P97.39	Cucumber	-	[41]
P97-1	Cucumber	-	[41]
P97-2	Cucumber	-	[41]
P97-6	Tomato	-	[41]
P97-20	Wheat	-	[41]
P97-26	Tomato	-	[41]
P97-27	Cucumber	-	[41]
P97-30	Wheat	-	[41]
PF36	Unknown	-	[63]
Pf-153	Tobacco	-	[80]
Q1-87	Wheat	-	[25]
Q2-87	Wheat	-	[81]
Q7-87	Wheat	-	[25]
Q12-87	Wheat	-	[25]
Q13-87	Wheat	-	[25]
Q37-87	Wheat	-	[25]
Q128-87	Wheat	-	[82]
S7-29	Tobacco	-	[39]
S7-46	Tobacco	-	[39]
S7-52	Tobacco	-	[39]
S7-42	Tobacco	-	[39]
S8-110	Tobacco	-	[39]
S8-130	Tobacco	-	[39]
S8-151	Tobacco	-	[40]
TM1A3	Tomato	-	[25]

TM1A4	Tomato	-	[83]
TM1B2	Tomato	-	[76]
53K-B2	Unknown	-	This study
Pseudomonas kilonensis			
520-20	Soil	-	[84]
Pseudomonas plecoglossicida			
PFCP1	Soil	-	[78]
Pseudomonas protegens[b]			
C6.2	Tobacco	+	[78]
C6-23	Tobacco	+	[78]
CHA0	Tobacco	+	[85]
K94-4	Tomato	+	[41]
K94-5	Tomato	+	[41]
K94-6	Cucumber	+	[25]
K94-30	Cucumber	+	[41]
K94-40	Cucumber	+	[41]
K94-41	Cucumber	+	[41]
M	Sunflower	+	This study
PF	Wheat	+	[86]
BRIP	Cyclops (water)	+	This study
Pf1	Tobacco	+	[25]
Pf-5	Cotton	+	[87]
Pf-68	Sunflower	+	[88]
Pf-100	Sunflower	+	[88]
PGNL1	Tobacco	+	[25]
PGNR1	Tobacco	+	[25]
PGNR2	Tobacco	+	[25]
S8-62	Tobacco	+	[78]
P6-1	Unknown	+	This study
Pseudomonas putida			
8176 NCBI	Milk	-	[89]
KB1	Soil	-	[90]
KT2440	Soil	-	[91]
LMG2257	Soil	-	[64]
Pseudomonas rhizospherae			
IH5	Rhizosphere grass	-	[92]
Pseudomonas sp.			
A506	Pear leaves	-	[93]
B13	Sewage	-	[94]
CMR12a	Cocoyam	+	[29]
DSS73	Soil	-	[95]
Jan	Apple blossom	-	[96]
Pseudomonas syringae			
ATCC 19310	Lilac	-	[97]
LMG 1247	Lilac	-	LMG collection
Rhodococcus sp.			
C125	River sediment	-	[98]
Sphingomonas herbicidovorans			
MH	Soil	-	[99]
Sphingomonas paucimobilis			
UT26	Soil	-	[100]
Staphylococcus aureus			
COL	Hospital	-	[101]
MW2	Human	-	[102]
RN4220	Laboratory strain		[103]
Staphylococcus epidermidis			
RP62A	Hospital	-	[104]
Streptomyces turgidiscabies			
Sy9103	Scab lesions	-	[105]
Xanthomonas campestris			

ATCC33913	Plant	-	[78]
Xenorhabdus bovienii			
13	*Steinernema* (Swiss soil)	+	This study
14	*Steinernema* (Swiss soil)	+	This study
25	*Steinernema* (Swiss soil)	+	This study
26	*Steinernema* (Swiss soil)	+	This study
27	*Steinernema* (Swiss soil)	+	This study
28	*Steinernema* (Swiss soil)	+	This study
29	*Steinernema* (Swiss soil)	+	This study
30	*Steinernema* (Swiss soil)	+	This study
31	*Steinernema* (Swiss soil)	+	This study
33	*Steinernema* (Swiss soil)	+	This study
35	*Steinernema* (Swiss soil)	+	This study
36	*Steinernema* (Swiss soil)	+	This study
37	*Steinernema* (Swiss soil)	+	This study
39	*Steinernema* (Swiss soil)	+	This study
44	*Steinernema* (Swiss soil)	+	This study
61	*Steinernema* (Swiss soil)	+	This study
64	*Steinernema* (Swiss soil)	+	This study
C	*Steinernema* (Swiss soil)	+	This study
D	*Steinernema* (Swiss soil)	+	This study
F	*Steinernema* (Swiss soil)	+	This study
J	*Steinernema* (Swiss soil)	+	This study
SS-2004	*Steinernema* (Swiss soil)	+	[106]
Xenorhabdus nematophila			
ATCC 19061	*Steinernema carpocapsae*	+	[106]

[a] If a plant is indicated, the respective strain has been isolated from its roots or rhizosphere

[b] *P. protegens* was recently proposed as an own species [26] and has been designated as *P. fluorescens* in previous publications
