# Supplementary material for: Evolutionary patchwork of an insecticidal toxin shared between plant-associated pseudomonads and the insect pathogens Photorhabdus and Xenorhabdus
Source: BMC Genomics. 2015 Aug 16;16(1):609. doi: 10.1186/s12864-015-1763-2 (PMC4542124; doi:10.1186/s12864-015-1763-2)
Supplement: Additional file 2: Table S2. — BLASTp analysis of P. protegens CHA0 Fit components against completed bacterial whole genome sequences. [file 12864_2015_1763_MOESM2_ESM.pdf]

Table S2 BLASTp analysis of *P. protegens* CHA0 Fit components against completed bacterial whole genome sequences.

Query	BLAST hit[a]	Product	Species	% aa identity (similarity)	Coverage	e-value
FitA	NP_930357.1	plu3125	*Photorhabdus luminescens* subsp. *laumondii* TTO1	69 (82)	98	0.0
	YP_001448573.1	RTX toxin transporter	*Vibrio harveyi* ATCC BAA-1116	59 (76)	97	0.0
	YP_003041907.1	RTX toxin ABC transporter	*Photorhabdus asymbiotica* subsp. *asymbiotica* ATCC 43949	59 (76)	88	0.0
	NP_928643.1	RTX toxin ABC transporter	*Photorhabdus luminescens* subsp. *laumondii* TTO1	58 (76)	88	0.0
	NP_762443.2	RTX toxin transporter	*Vibrio vulnificus* CMCP6	58 (76)	88	0.0
FitB	NP_930358.1	plu3126	*Photorhabdus luminescens* subsp. *laumondii* TTO1	65 (83)	97	0.0
	YP_001448572.1	VIBHAR_06454	*Vibrio harveyi* ATCC BAA-1116	52 (71)	94	3e-150
	YP_002923190.1	ABC transporter	Candidatus *Hamiltonella defensa* 5AT	50 (71)	94	2e-137
	YP_070687.1	RTX toxin ABC transporter	*Yersinia pseudotuberculosis* IP 32953	49 (67)	98	6e-113
	YP_004298409.1	hemolysin transport protein	*Yersinia enterocolitica subsp. palearctica* 105.5R	47 (66)	98	4e-109
FitC	NP_930359.1	plu3127	*Photorhabdus luminescens* subsp. *laumondii* TTO1	73 (84)	98	0.0
	ZP_01956307.1	toxin secretion transporter	*Vibrio cholerae* MZO-3	58 (74)	97	0.0
	NP_762445.1	RTX toxin transporter	Vibrio vulnificus CMCP6	59 (74)	97	
	NP_928641.1	plu1331	*Photorhabdus luminescens* subsp. *laumondii* TTO1	57 (74)	99	0.0
	YP_004298408.1	putative toxin transport protein	*Yersinia enterocolitica* subsp. *palearctica* 105.5R	58 (72)	98	0.0
FitD	NP_931332.1	Mcf protein	*Photorhabdus luminescens* subsp. *laumondii* TTO1	73 (83)	100	0.0
	YP_003042199.1	MCF toxin	*Photorhabdus asymbiotica* subsp. *asymbiotica* ATCC 43949	71 (81)	97	0.0
	NP_930360.1	plu3128	*Photorhabdus luminescens* subsp. *laumondii* TTO1	67 (81)	69	0.0
	YP_003712268.1	XNC1_2028	*Xenorhabdus nematophila* ATCC 19061	64 (78)	69	0.0
	YP_003468304.1	Mcf protein	*Xenorhabdus bovienii* SS-2004	69 (79)	74	0.0
	YP_003712501.1	XNC1_2265	*Xenorhabdus nematophila* ATCC 19061	65 (76)	63	0.0

	EGU44028.1	Mcf2	*Vibrio splendidus* ATCC 33789	29 (48)	65	0.0
FitE	ZP_10648180.1	PSEBR_a511	*Pseudomonas brassicacearum* subsp. *brassicacearum* NFM421	62 (79)	89	4e-159
	YP_346224.1	Type I secretion outer membrane protein, TolC	*Pseudomonas fluorescens* Pf0-1	61 (78)	90	7e-159
	YP_257681.1	TolC family type I secretion outer membrane protein	*Pseudomonas protegens* Pf-5	61 (78)	90	2e-156
	EGH31280.1	Type I secretion outer membrane protein, TolC	*Pseudomonas syringae* pv. *japonica* str. M301072	62 (79)	90	5e-155
	ZP_07003042.1|	Type I secretion outer membrane protein, TolC precursor	*Pseudomonas savastanoi* pv. *savastanoi* NCPPB 3335	63 (79)	88	1e-154
FitF	YP_285112.1	PAS/PAC sensor hybrid histidine kinase	*Dechloromonas aromatica* RCB (beta)	41 (57)	48	1e-106
	YP_005041177.1	Hybrid sensor histidine kinase	*Azospirillum lipoferum* 4B	38 (60)	61	3e-100
	YP_865134.1	multi-sensor hybrid histidine kinase	*Magnetococcus marinus* MC-1	37 (58)	49	4e-98
	YP_004514611.1	PAS domain-containing protein	*Thiocystis violascens* DSM 198	40 (58)	66	6e-98
	YP_005050479.1	multi-sensor hybrid histidine kinase	*Methylomonas methanica* MC09]	34 (54)	67	9e-98
FitG	YP_004499875.1	LysR family transcriptional regulator	*Serratia* sp. AS12	45 (65)	98	3e-79
	YP_348645.1	LysR family transcriptional regulator	*Pseudomonas fluorescens* Pf0-1	45 (64)	95	7e-79
	YP_260488.1	LysR family transcriptional regulator	*Pseudomonas protegens* Pf-5	46 (63)	95	6e-76
	ZP_07775323.1	LysR family transcriptional regulator	*Pseudomonas fluorescens* WH6	45 (61)	97	2e-73
	YP_001007048.1	LysR family transcriptional regulator	*Yersinia enterocolitica* subsp. *enterocolitica* 8081	42 (64)	97	2e-73
FitH	YP_005025997.1	response regulator receiver modulated metal dependent phosphohydrolase	*Vibrio* sp. EJY3 (gamma)	54 (76)	36	0.0
	NP_441314.1	regulatory components of sensory transduction system	*Synechocystis* sp. PCC 6803 (cyano)	53 (74)	36	9e-33
	YP_002954478.1	response regulator receiver protein	*Desulfovibrio magneticus* RS-1 (delta)	47 (62)	44	2e-32
	YP_006416106.1	response regulator containing a CheY-like receiver domain and an HD-GYP domain	*Thiocystis violascens* DSM 198 (gamma)	52 (72)	35	1e-31
	YP_421203.1	response regulator	*Magnetospirillum magneticum* AMB-1 (alpha)	52 (68)	39	5e-31

A total of 1234 completed genomic sequences were included in BLASTp searches. Highly identical sequences (≤ 2% divergence) within the same species were omitted.
