# Supplementary material for: Evolutionary patchwork of an insecticidal toxin shared between plant-associated pseudomonads and the insect pathogens Photorhabdus and Xenorhabdus
Source: BMC Genomics. 2015 Aug 16;16(1):609. doi: 10.1186/s12864-015-1763-2 (PMC4542124; doi:10.1186/s12864-015-1763-2)
Supplement: Additional file 3: Figure S1. — Insecticidal activity correlates with presence of the fitD gene. [file 12864_2015_1763_MOESM3_ESM.pdf]

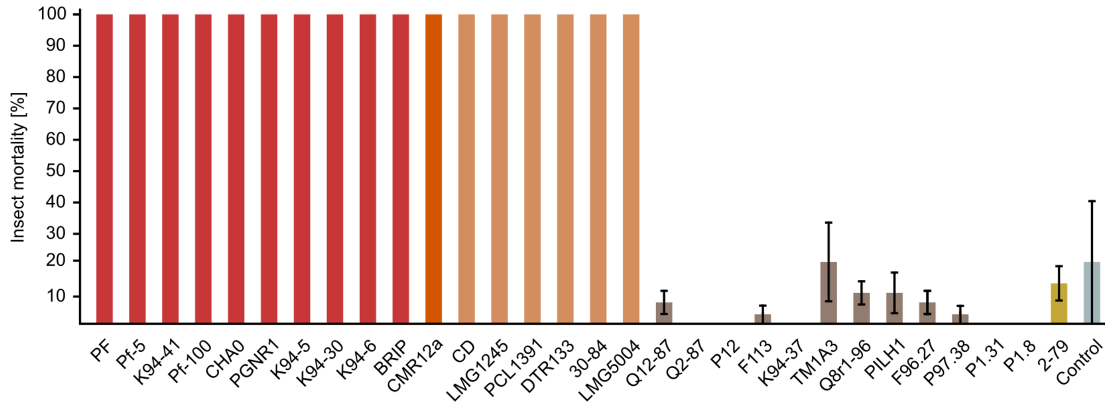

Figure S1 Insecticidal activity correlates with presence of the *fitD* gene. Insect mortality of *G. mellonella* larvae four days after injection of 4×10^4 cells of 17 Fit producing and 13 non-producing *Pseudomonas* strains. Each strain was tested on a total of 30 larvae (five replicate plates with six larvae per plate). Bars show average of insect mortality for each strain. Error bars show standard error of the mean. Fit^+ strains were significantly different from Fit^- based on Wilcoxon rank sum test grouped by Fit^+ and Fit^- strains ($P< 0.05$).
